# Antibiotic-resistant *Escherichia coli* from retail poultry meat with different antibiotic use claims

**DOI:** 10.1186/s12866-018-1322-5

**Published:** 2018-11-03

**Authors:** Gregg S. Davis, Kara Waits, Lora Nordstrom, Heidi Grande, Brett Weaver, Katerina Papp, Joseph Horwinski, Benjamin Koch, Bruce A. Hungate, Cindy M. Liu, Lance B. Price

**Affiliations:** 1 George Washington University Milken Institute School of Public Health, Washington, DC USA; 2 Pathogen Genomics Division, Translational Genomics Research Institute, Flagstaff, AZ USA; 3 The Biodesign Center for Fundamental and Applied Microbiomics, Center for Evolution and Medicine, School of Life Sciences, Arizona State University, Tempe, AZ USA; 4 Department of Civil and Environmental Engineering and Construction, University of Las Vegas, Las Vegas, NV USA; 5 Division of Hydrologic Sciences, Desert Research Institute, Las Vegas, NV USA; 6 Center for Ecosystem Science and Society and Department of Biological Sciences, Northern Arizona University, Flagstaff, AZ USA

**Keywords:** Antibiotic, Poultry, Organic, Raised without antibiotics, Conventional, *E. coli*, Multidrug resistance, Antibiotic

## Abstract

**Background:**

We sought to determine if the prevalence of antibiotic-resistant *Escherichia coli* differed across retail poultry products and among major production categories, including organic, “raised without antibiotics”, and conventional.

**Results:**

We collected all available brands of retail chicken and turkey—including conventional, “raised without antibiotic”, and organic products—every two weeks from January to December 2012. In total, *E. coli* was recovered from 91% of 546 turkey products tested and 88% of 1367 chicken products tested. The proportion of samples contaminated with *E. coli* was similar across all three production categories. Resistance prevalence varied by meat type and was highest among *E. coli* isolates from turkey for the majority of antibiotics tested. In general, production category had little effect on resistance prevalence among *E. coli* isolates from chicken, although resistance to gentamicin and multidrug resistance did vary. In contrast, resistance prevalence was significantly higher for 6 of the antibiotics tested—and multidrug resistance—among isolates from conventional turkey products when compared to those labelled organic or “raised without antibiotics”. *E. coli* isolates from chicken varied strongly in resistance prevalence among different brands within each production category.

**Conclusion:**

The high prevalence of resistance among *E. coli* isolates from conventionally-raised turkey meat suggests greater antimicrobial use in conventional turkey production as compared to “raised without antibiotics” and organic systems. However, among *E. coli* from chicken meat*,* resistance prevalence was more strongly linked to brand than to production category, which could be caused by brand-level differences during production and/or processing, including variations in antimicrobial use.

**Electronic supplementary material:**

The online version of this article (10.1186/s12866-018-1322-5) contains supplementary material, which is available to authorized users.

## Background

Antibiotic use in food-animal production has major implications for public health. The routine use of antibiotics on farms—regardless of their indication—selects for and maintains a reservoir of resistant bacteria capable of causing human disease or of passing mobile resistance determinants to human pathogens [1–6]. The relevance of agricultural use of antibiotics to human health is underscored by the fact that 62% of the 34.3 million pounds of antibiotics sold or distributed for use in US food-animal production during 2015 were considered “medically important” to human health [7].

*Escherichia coli* is a common inhabitant of the vertebrate intestinal tract and a frequent microbial contaminant of retail meat products. As part of the National Antimicrobial Resistance Monitoring System (NARMS), the U.S. Food and Drug Administration monitors antibiotic resistance trends among foodborne *E. coli*. Resistance has been increasing among both clinical and foodborne *E. coli* and, from 1950 to 2002, resistance increased at a faster rate among livestock isolates than it did among human clinical isolates [8]. Despite the increasing public health concerns over antibiotic use in food-animal production, the amount of antibiotics sold or distributed for use in food-producing animals increased every year between 2009 to 2015 [7]. More recently, plasmid-mediated colistin resistance has been detected in livestock, on retail meat products, and in humans [9–11]. Colistin is one of the few antibiotics that can be used to treat carbapenem-resistant infections [12]; however, it is also used for disease prevention and growth promotion in livestock production in some countries [10–14]. The increasing prevalence of colistin resistance globally poses a significant threat to the safety of the world’s food supply.

Livestock production practices and retail meat labels can vary with regards to antibiotic use. For example, antibiotic use regulations are more stringent for animals that are slaughtered for meat labelled “raised without antibiotics” (RWA) and “organic” than those that are slaughtered for conventional products. However, even RWA and organic standards allow for some antibiotic use. For example, the organic standard for poultry begins on “second day of life” (USDA CFR Title 7 §205.236) and thus does not restrict antibiotic use prior to that stage. RWA standards span from “birth to harvest” [15]. Therefore, both RWA and organic standards allow for *in ovo* (in egg) antibiotic injections concurrently with vaccinations.

Consumer awareness about the relationship between antibiotic use in food-animals and antibiotic resistance has lead, in part, to increased market demand for products labelled RWA and organic [16]. Bacteria recovered from conventionally raised poultry are generally resistant to more antibiotics, and are more likely to be multidrug resistant, than are those isolated from products raised without antibiotics or organically [2–6], although some exceptions have been reported [17, 18]. In the current study, we systematically sampled retail poultry products every two weeks over the course of an entire calendar year and asked whether the prevalence of antibiotic-resistant *E. coli* differed by source and production category. In addition, we characterized brand-specific differences among *E. coli* isolates contaminating retail chicken products, making this study unique in its sampling intensity and its consideration of brand-level differences in resistance prevalence.

## Methods

### Sample collection and processing

Samples were collected [19] and processed [17] as described previously. Briefly, all available brands of retail chicken and turkey products were collected every two weeks from nine stores, representing each of the major grocery chains, in Flagstaff, AZ from January 2012 to December 2012. Data collected from each product included: store name, sell-by date, brand name, cut type, P-number (production plant number), special labels (e.g., organic, natural, raised without antibiotics, etc.) and a photo of the package. Products were refrigerated at 4 °C and processed no later than one day past their sell-by date.

A single *E. coli* isolate was randomly selected from each package of retail poultry as described previously [17]. To be confirmed as *E. coli,* isolates had to exhibit characteristic growth on VRBA+MUG and CHROMagar plates and be *uidA*-positive by qPCR in a confirmatory assay [17] using primers: uidA_F: 5’-CGTATCACHGTTTGTGTGAACAA-3′, uidA_R: 5’-GGATTCACYACTTGCAAAGTCC-3′, and uidA_probe: (VIC) 5’-AACTGGCAGACTATCC-3′.

### Susceptibility testing

Each isolate was tested for susceptibility to amikacin, ampicillin, ampicillin-sulbactam, cefazolin, cefoxitin, ceftriaxone, ciprofloxacin, gentamicin, imipenem, nalidixic acid, tetracycline, and trimethoprim-sulfamethoxazole. Susceptibility was determined by the disk diffusion method in accordance with 2017 Clinical Laboratory Standards Institute M100, 27th edition guidelines and breakpoints [20]. Isolates classified as “intermediate” were grouped with “resistant” isolates for all statistical analyses. Multidrug resistance was defined as resistance to three or more classes of antibiotics.

### Extended spectrum β-lactamase (ESBL) confirmatory test

ESBL phenotype was confirmed by assessing the ability of clavulanic acid (10 μg) to inhibit the activity of cefotaxime (30 μg) and ceftazidime (30 μg) in a standard combination disk diffusion test (BD Diagnostic Systems, Sparks, MD). If the addition of clavulanic acid increased the zone of inhibition by ≥5 mm when compared to the drug alone, the isolate was defined as an ESBL-producer [20].

### Statistical analyses

Two-tailed Fisher’s exact tests were used to compare resistance prevalence across categories. The threshold for statistical significance was α = 0.05; no corrections were made for multiple comparisons. All analyses were implemented in the software package R version 3.0.1 [21].

## Results

### Sample collection

During 2012, 1367 packages of chicken and 546 packages of turkey meat were purchased from grocery stores in Flagstaff, Arizona. These included 1214 conventional products, 255 organic products, and 444 RWA products. Thirty-four brands were sampled, including 18 conventional brands, 8 organic brands, and 8 RWA brands (Additional file 1: Table S1).

### *E. coli* prevalence

Chicken products (87.6%) were less likely to be contaminated with *E. coli* than were turkey products (90.7%), but the difference was not statistically significant (*P = 0.0575*). Within each meat type, the prevalence of *E. coli* contamination did not vary by production category. (Additional file 2: Figure S1).

### Antibiotic resistance among *E. coli* isolates

Resistance was detected to 10 of the 12 antibiotics tested. None of the isolates were resistant to amikacin or imipenem. Nine isolates—5 turkey and 4 chicken—displayed phenotypically confirmed ESBL production.

**Fig. 1:**
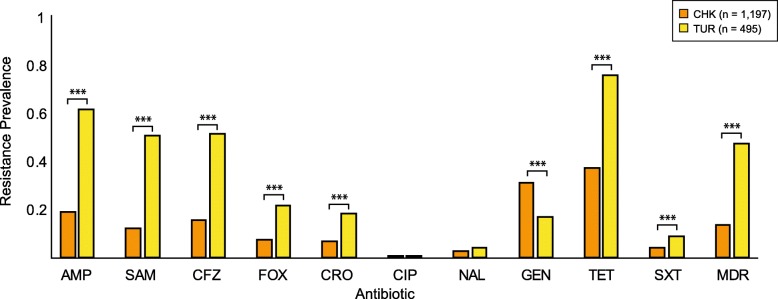
Prevalence of antibiotic resistance among *E. coli* isolates contaminating retail chicken and turkey. Each isolate was tested against: ampicillin (AMP), ampicillin-sulbactam (SAM), cefazolin (CFZ), cefoxitin (FOX), ceftriaxone (CRO), ciprofloxacin (CIP), nalidixic acid (NAL), gentamicin (GEN), tetracycline (TET), trimethoprim-sulfamethoxazole (SXT), amikacin (AMK), and imipenem (IPM). Multidrug resistance (MDR) was defined as resistance to three or more classes of antibiotics. None of the isolates tested were resistant to amikacin (AMK) or imipenem (IPM), which are excluded from the figure. The prevalence of antibiotic resistance was compared among meat types using the two-tailed Fisher’s exact test. (*** *P < 0.001*)

Resistance prevalence varied by meat type and, for 8 individual antibiotics and multidrug resistance, the differences were statistically significant (Fig. 1). Resistance prevalence was highest among *E. coli* isolates recovered from turkey meat for nearly all antibiotics tested, with greater than 50% of the isolates displaying resistance to ampicillin (62%), ampicillin-sulbactam (51%), cefazolin (52%), and/or tetracycline (76%) (Fig. 1). Furthermore, 48% of the *E. coli* isolates from turkey were multidrug resistant.

### Antibiotic resistance by production category

**Fig. 2:**
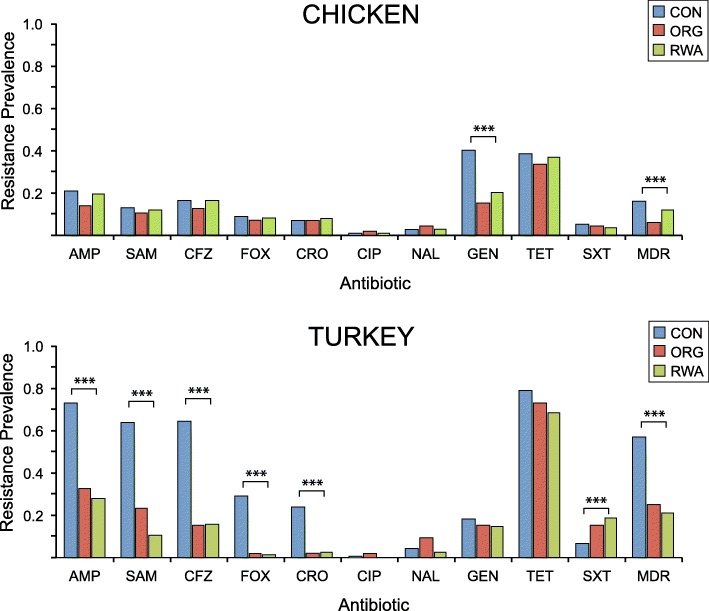
Antibiotic resistance prevalence among *E. coli* isolates contaminating retail poultry meats raised conventionally (CON), organically (ORG), or “raised without antibiotics” (RWA). Each isolate was tested against: ampicillin (AMP), ampicillin-sulbactam (SAM), cefazolin (CFZ), cefoxitin (FOX), ceftriaxone (CRO), ciprofloxacin (CIP), nalidixic acid (NAL), gentamicin (GEN), tetracycline (TET), trimethoprim-sulfamethoxazole (SXT), amikacin (AMK), and imipenem (IPM), which are excluded from the figure. Multidrug resistance (MDR) was defined as resistance to three or more classes of antibiotics. None of the isolates tested were resistant to amikacin (AMK) or imipenem (IPM). The prevalence of antibiotic resistance was compared among production categories using the two-tailed Fisher’s exact test. (*** *P < 0.001*, * *P < 0.05*)

To assess the impact of antibiotic use, as reflected by label claims, comparisons were made across production categories within each meat type (Fig. 2). Production category had minimal impact on resistance prevalence among chicken isolates, only gentamicin (*P < 0.001*) and multidrug resistance (*P < 0.001*) varied significantly across production categories. In contrast, *E. coli* isolates from turkey exhibited significant variation in resistance prevalence to 6 of the antibiotics studied, and to multidrug resistance, across production categories (Fig. 2). Interestingly, trimethoprim-sulfamethoxazole resistance prevalence was lowest among turkey isolates from conventional products and highest among RWA products. Lastly, tetracycline resistance was prevalent across production categories in all meat types, ranging from 34% (organic chicken) to 78% (conventionally raised turkey).

### Antibiotic resistance across retail chicken brands

**Fig. 3:**
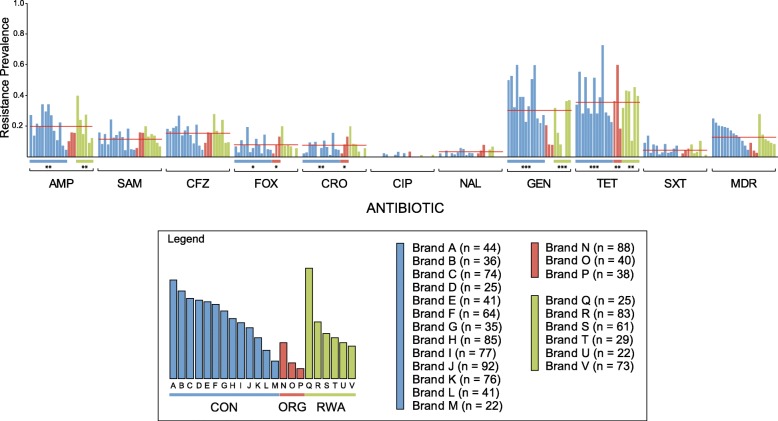
Antibiotic resistance prevalence among *E. coli* isolates contaminating retail brands of chicken meat. Each bar represents a unique brand of chicken, bars are color coded by production category, i.e., conventionally raised (CON), organically raised (ORG), or “raised without antibiotics” (RWA). The horizontal red line indicates the average resistance prevalence across all brands and categories for each antibiotic. Each isolate was tested against: ampicillin (AMP), ampicillin-sulbactam (SAM), cefazolin (CFZ), cefoxitin (FOX), ceftriaxone (CRO), ciprofloxacin (CIP), nalidixic acid (NAL), gentamicin (GEN), tetracycline (TET), trimethoprim-sulfamethoxazole (SXT), amikacin (AMK), and imipenem (IPM). Multidrug resistance (MDR) was defined as resistance to three or more classes of antibiotics. None of the isolates tested were resistant to amikacin (AMK) or imipenem (IPM), which are excluded from the figure. The prevalence of antibiotic resistance, and MDR, was compared across all brands within each production category using the two-tailed Fisher’s exact test. All brands were included in the statistical analysis (Additional file 1: Table S1); however, only brands with more than 20 isolates are included in the figure. (*** *P < 0.001*, **P < 0.01, * *P < 0.05*)

The large number of chicken brands tested allowed for brand-to-brand comparisons within each production category. The prevalence of antibiotic resistance differed among brands for 6 of the antibiotics tested (Fig. 3). Isolates from conventional brands differed significantly in their resistance to ampicillin (*P = 0.003*), cefazolin (*P = 0.036*), cefoxitin (*P = 0.015*), ceftriaxone (*P = 0.008*), gentamicin (*P < 0.001*), and tetracycline (*P < 0.001*). Isolates from organic brands exhibited variable resistance to cefoxitin (*P = 0.027*), ceftriaxone (*P = 0.027*), and tetracycline (*P = 0.003*). There was significant variability among ampicillin (*P = 0.015*), gentamicin (*P < 0.001*), and tetracycline (*P = 0.003*) resistance among isolates from RWA brands. The prevalence of multidrug resistance did not differ significantly across brands.

## Discussion

Resistance was detected to the majority (9/12) of antibiotics tested but varied by meat type. Resistance prevalence was highest among *E. coli* isolated from turkey for nearly all antibiotics tested, with greater than half of all isolates resistant to classes of antibiotics that are important in human medicine [7]. Furthermore, 50% of the *E. coli* from turkey were multidrug resistant. These findings suggest that there are greater antibiotic selective pressures in turkey production than in chicken production. However, U.S. producers are not required to publicly report species-specific data on antimicrobial use, making it impossible to draw firm conclusions linking on-farm antibiotic use to antibiotic-resistance. From the consumer’s perspective, our results indicate that nearly half of all packages of retail turkey were contaminated with multidrug resistant *E. coli.*

Increasing awareness about the risks associated with antibiotic use in food-animal production has led to a shift in consumer perceptions and investments in organic products [22, 23]. Consumers may choose organic products, in part, to reduce their exposure to antibiotic-resistant bacteria. And, previous studies have shown that organic poultry is less likely to be contaminated with drug-resistant *Campylobacter*, *Salmonella,* and *Enterococcus* species when compared to conventional products [2–6, 24]. We detected significant variation across production categories among *E. coli* from ground turkey for six antibiotics and, in the majority of cases, resistance was most common among isolates from conventionally raised turkeys. However, production category had little influence on resistance prevalence among *E. coli* isolates from chicken—suggesting similar selective pressures across all three categories for the majority of antibiotics tested. One notable exception was gentamicin resistance, which was highest among isolates from conventionally raised chicken. Gentamicin is commonly added to *in ovo* vaccines [25], and previous studies have shown that *in ovo* injection of antibiotics can be a major driver for antibiotic-resistant bacterial contamination on retail poultry [26]. Thus, use of gentamicin-supplemented vaccines may explain why resistance to this antibiotic is prevalent among isolates from conventionally raised chicken products.

Counterintuitively, the prevalence of trimethoprim-sulfamethoxazole resistance was highest among isolates from organic turkey and lowest among those from conventional products (Fig. 2). This may be due to increased supplementation of animal feeds with metals, such as zinc and copper, which are used to promote growth and reduce disease but may also select for, tetracycline, suflanomide and multidrug resistance [27–30]. Tetracycline resistance was high across all production categories, even though antibiotic use is restricted in RWA and organic production. Previous studies have demonstrated that tetracycline resistance can persist long after the cessation of tetracycline use [31]. These findings highlight the complexity of antibiotic resistance and demonstrate that in some cases resistance can linger long after antibiotic use has ceased.

Brand was a strong predictor of resistance among *E. coli* from chicken products, suggesting that company-specific production policies may actually outweigh USDA antibiotic use regulations. These regulations are largely restricted to the broiler grow-out period that spans from hatching to slaughter; however, factors before and after the grow-out period may affect the antibiotic susceptibility of *E. coli* contaminating retail meat products. In poultry production, antibiotic use in breeder and parent flocks is not regulated under USDA organic or RWA regulations. Thus, vertical transmission of antibiotic-resistant isolates through the production pyramid from grandparent stocks, to parent stocks, and to broiler flocks may affect contaminants on retail products [32–37]. Furthermore, previous work has shown that *in ovo* (in egg) injection of antibiotics can be a major driver for antibiotic-resistant bacteria on retail poultry and antibiotic-resistant human infections [26].

Downstream of grow-out, cross-contamination during broiler slaughter and processing could blur the microbial quality of the three production categories. Thus, company-level information regarding antibiotic use upstream of grow-out as well as downstream slaughter protocols could help reveal the primary factors affecting the prevalence of antibiotic-resistant bacteria on retail poultry products.

The production history of the grow-out facility itself may also have a significant bearing on antibiotic resistance. Broiler barns, or farms, that have been converted from conventional to organic production may still harbor antibiotic-resistant bacteria in the litter, albeit at reduced levels [24]. Organic standards prohibit the sale of antibiotic-treated animals under the organic label, but in the absence of approved organic therapeutics, antibiotics are mandated for the treatment of sick animals when necessary (e.g., during a disease outbreak). Yet, there are no specific guidelines for decontaminating the production facilities after such treatments. Bacteria that are shed during treatment could potentially persist in the environment, seeding subsequent flocks and herds. Similarly, management of multiple production categories on the same farm presents opportunities for antibiotic-resistant bacteria to spread from treated to non-treated animals.

Here we have shown that, while organically raised chicken and turkey are generally less likely to be contaminated with antibiotic-resistant *E. coli,* the distinctions among the three production categories are not as sharp as one might expect. Instead, the data from chicken suggest that brand is a more powerful predictor of antibiotic-resistant *E. coli* than is production category. However, few companies publish their production policies in a manner that can be used to inform consumer choice, and there is no federal effort to monitor and publish this information. Increased transparency regarding antibiotic use practices throughout the production system, spanning from breeding to grow-out would enable consumers to make more informed purchasing decisions.

## Conclusions

The high prevalence of resistance among *E. coli* isolates from conventionally-raised turkey meat suggests that there is greater antimicrobial use in conventional turkey production as compared to RWA and organic systems. In contrast, there were few differences in antibiotic resistance prevalence among *E. coli* isolates across categories of chicken production. Instead, resistance prevalence was more strongly linked to brand, which could be caused by brand-level differences during production and/or processing, including variations in antimicrobial use*.*

## Additional files

Additional file 1: **Table S1.** Proportion of retail chicken (a) and turkey (b) samples contaminated with *E. coli*. (DOCX 87 kb)

Additional file 2: **Figure S1.** Proportion of retail chicken and turkey meat samples contaminated with *E. coli.* Within each meat type, samples were stratified by production category, i.e., conventional (CON), organic (ORG), or “raised without antibiotics” (RWA). (PDF 829 kb)

## Abbreviations

AMK: Amikacin
AMP: Ampicillin
CFZ: Cefazolin
CIP: Ciprofloxacin
CON: Conventional
CRO: Ceftriaxone
FOX: Cefoxitin
GEN: Gentamicin
IPM: Imipenem
MDR: Multidrug resistance (i.e., resistance to three or more classes of antibiotics)
NAL: Nalidixic acid
ORG: Organic
RWA: Raised without antibiotics
SAM: Ampicillin-sulbactam
SXT: Trimethoprim-sulfamethoxazole
TET: Tetracycline
