# Supplementary material for: Antibiotic-resistant Escherichia coli from retail poultry meat with different antibiotic use claims
Source: BMC Microbiol. 2018 Nov 3;18:174. doi: 10.1186/s12866-018-1322-5 (PMC6215666; doi:10.1186/s12866-018-1322-5)
Supplement: Supplementary file 1 — Table S1. Proportion of retail chicken (a) and turkey (b) samples contaminated with E. coli. (DOCX 87 kb) [file 12866_2018_1322_MOESM1_ESM.docx]

Table S1. Proportion of retail chicken (a) and turkey (b) samples contaminated with *E. coli*.

(a) Chicken

| Brand^a^ | Production Category^b^ | Packages of Chicken Purchased | Number of Packages Contaminated with *E. coli* | Proportion of Samples Contaminated |
| --- | --- | --- | --- | --- |
| Brand A_C_ | CON | 48 | 44 | 0.917 |
| Brand B_C_ | CON | 38 | 36 | 0.947 |
| Brand C_C_ | CON | 88 | 74 | 0.841 |
| Brand D_C_ | CON | 26 | 25 | 0.962 |
| Brand E_C_ | CON | 46 | 41 | 0.891 |
| Brand F_C_ | CON | 66 | 64 | 0.970 |
| Brand G_C_ | CON | 42 | 35 | 0.833 |
| Brand H_C_ | CON | 90 | 85 | 0.944 |
| Brand I_C_ | CON | 92 | 77 | 0.837 |
| Brand J_C_ | CON | 110 | 92 | 0.836 |
| Brand K_C_ | CON | 92 | 76 | 0.826 |
| Brand L_C_ | CON | 46 | 41 | 0.891 |
| Brand M_C_ | CON | 24 | 22 | 0.917 |
|  | **CON** | **808** | **712** | **0.881** |
|  |  |  |  |  |
| Brand N_C_ | ORG | 91 | 88 | 0.967 |
| Brand O_C_ | ORG | 40 | 25 | 0.625 |
| Brand P_C_ | ORG | 44 | 38 | 0.864 |
| Brand W_C_ | ORG | 20 | 20 | 1.000 |
| Brand X_C_ | ORG | 2 | 2 | 1.000 |
|  | **ORG** | **197** | **173** | **0.878** |
|  |  |  |  |  |
| Brand Q_C_ | RWA | 34 | 25 | 0.735 |
| Brand R_C_ | RWA | 84 | 83 | 0.988 |
| Brand S_C_ | RWA | 90 | 61 | 0.678 |
| Brand T_C_ | RWA | 32 | 29 | 0.906 |
| Brand U_C_ | RWA | 22 | 22 | 1.000 |
| Brand V_C_ | RWA | 80 | 73 | 0.913 |
| Brand Y_C_ | RWA | 20 | 19 | 0.950 |
|  | **RWA** | **362** | **312** | **0.862** |
|  |  |  |  |  |
| **TOTAL** |  | **1367** | **1197** | **0.876** |

(b) Turkey

| Brand | Category^b^ | Packages of Turkey Purchased | Number of Packages Contaminated with *E. coli* | Proportion of Samples Contaminated |
| --- | --- | --- | --- | --- |
| Brand A_T_ | CON | 14 | 8 | 0.571 |
| Brand B_T_ | CON | 292 | 269 | 0.921 |
| Brand C_T_ | CON | 22 | 22 | 1.000 |
| Brand D_T_ | CON | 46 | 42 | 0.913 |
| Brand E_T_ | CON | 32 | 26 | 0.813 |
|  | **CON** | **406** | **367** | **0.904** |
|  |  |  |  |  |
| Brand F_T_ | ORG | 2 | 2 | 1.000 |
| Brand G_T_ | ORG | 48 | 42 | 0.875 |
| Brand H_T_ | ORG | 8 | 8 | 1.000 |
|  | **ORG** | **58** | **52** | **0.893** |
|  |  |  |  |  |
| Brand I_T_ | **RWA** | **82** | **76** | **0.929** |
|  |  |  |  |  |
| **TOTAL** |  | **546** | **495** | **0.907** |

^a^ C = chicken, T = turkey

^b^ CON = conventional, ORG = organic, RWA = raised without antibiotics
