# Supplementary figures and images for: Antibiotic-resistant Escherichia coli from retail poultry meat with different antibiotic use claims
Source: BMC Microbiol. 2018 Nov 3;18:174. doi: 10.1186/s12866-018-1322-5 (PMC6215666; doi:10.1186/s12866-018-1322-5)

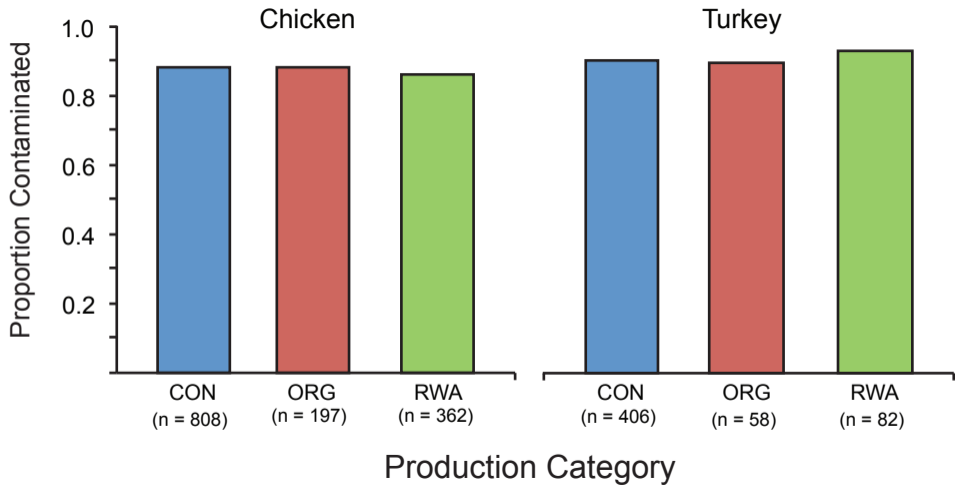

Supplement: Supplementary file 2 — Figure S1. Proportion of retail chicken and turkey meat samples contaminated with E. coli. Within each meat type, samples were stratified by production category, i.e., conventional (CON), organic (ORG), or “raised without antibiotics” (RWA). (PDF 829 kb) [file 12866_2018_1322_MOESM2_ESM.pdf]
